# Smartphone automated motor and speech analysis for early detection of Alzheimer's disease and Parkinson's disease: Validation of TapTalk across 20 different devices

**DOI:** 10.1002/dad2.70025

**Published:** 2024-10-23

**Authors:** Renjie Li, Guan Huang, Xinyi Wang, Katherine Lawler, Lynette R. Goldberg, Eddy Roccati, Rebecca J. St George, Mimieveshiofuo Aiyede, Anna E. King, Aidan D. Bindoff, James C. Vickers, Quan Bai, Jane Alty

**Affiliations:** ^1^ Wicking Dementia Research and Education Centre University of Tasmania Hobart Tasmania Australia; ^2^ School of ICT University of Tasmania Hobart Tasmania Australia; ^3^ School of Allied Health Human Services and Sport La Trobe University Melbourne Victoria Australia; ^4^ School of Psychological Sciences University of Tasmania Hobart Tasmania Australia; ^5^ School of Medicine University of Tasmania Hobart Tasmania Australia; ^6^ Neurology Department Royal Hobart Hospital Hobart Tasmania Australia

**Keywords:** biomarkers, dementia, Mediapipe, motor–cognitive, preclinical

## Abstract

**INTRODUCTION:**

Smartphones are proving useful in assessing movement and speech function in Alzheimer's disease and other neurodegenerative conditions. Valid outcomes across different smartphones are needed before population‐level tests are deployed. This study introduces the TapTalk protocol, a novel app designed to capture hand and speech function and validate it in smartphones against gold‐standard measures.

**METHODS:**

Twenty different smartphones collected video data from motor tests and audio data from speech tests. Features were extracted using Google Mediapipe (movement) and Python audio analysis packages (speech). Electromagnetic sensors (60 Hz) and a microphone acquired simultaneous movement and voice data, respectively.

**RESULTS:**

TapTalk video and audio outcomes were comparable to gold‐standard data: 90.3% of video, and 98.3% of audio, data recorded tapping/speech frequencies within ± 1 Hz of the gold‐standard measures.

**DISCUSSION:**

Validation of TapTalk across a range of devices is an important step in the development of smartphone‐based telemedicine and was achieved in this study.

**Highlights:**

- TapTalk evaluates hand motor and speech functions across a wide range of smartphones.
- Data showed 90.3% motor and 98.3% speech accuracy within +/–1 Hz of gold standards.
- Validation advances smartphone‐based telemedicine for neurodegenerative diseases.

## 1 INTRODUCTION

Neurodegenerative disorders pose significant challenges to health‐care systems worldwide.^1^ The prevalence and costs of these disorders are rapidly rising and the two most common are Alzheimer's disease (AD) and Parkinson's disease (PD).^2^, ^3^ There is an urgent need for objective precise measures that can detect these disorders and can be used away from a clinic at the population level.^4^ There is already a range of online methods to collect cognitive data unsupervised,^5^ but cognitive decline occurs relatively late in the disease course of AD and PD.^6^ In contrast, subtle dysfunction of motor and speech functions are early indicators and continue to progress throughout the disease.^7^, ^8^ Movement features of AD and PD include slowed gait,^9^ less rhythmic upper limb movements,^10^ tremors,^11^ and alterations of fine motor skills in the hands.^7^, ^8^, ^12^ Speech markers include alterations in articulation, fluency, voice, and language expression.^13^, ^14^ Precisely measuring such motor and speech features thus holds substantial promise for early detection and monitoring of these neurodegenerative conditions.^15^, ^16^, ^17^

The critical importance of automated remote motor‐ and speech analysis for early detection and monitoring^10^, ^18^ is increasingly being recognized and there has been a boom in the number of publications evaluating these methods in AD and PD over the last decade.^19^ Emerging research indicates that fine motor control, including hand and speech movements, is sensitive to early AD pathology.^20^, ^21^, ^22^ A specialized test can help detect these early signs, facilitating earlier intervention. Meanwhile, the wide reach of smartphones has made remote population‐level assessments feasible as 68% of the population globally own a smartphone, including the majority of adults aged > 60.^23^ Thus, a smartphone‐based application that automates motor and speech analysis would provide a tool with wide reach and impact, holding significant capacity to transform epidemiological studies and clinical trials and provide remote monitoring in a home environment.^17^

It is important to acknowledge that the quality of the video and audio data (needed to estimate motor and speech function) collected from different smartphone devices may vary.^24^ No previous studies have compared the validity of video and audio data collected across different smartphone devices to detect subtle changes in motor and speech function. This is an important gap to address as there is currently a range of smartphones being used in studies and ever‐increasing interest in the automated online analysis of data collected through smartphones.

We have developed TapTalk, a smartphone‐based self‐administered application that records hand motor video data (“Tap”), and speech‐like audio data (“Talk”), and then applies advanced analytics to extract a range of features such as rhythm and frequency.^25^ This pilot study aimed to outline the 2 minute test protocol and validate TapTalk video‐ and audio‐recorded data, from a range of smartphone models against established gold‐standard measures. This will be the first pilot study to evaluate the agreement between video and audio data extracted from different smartphone devices and data collected with gold‐standard measures. It has wider ramifications for other studies that require benchmarking of the validity of data collected through these specific devices.

## 2 METHODS

### 2.1 Study participants

Thirty‐three participants, recruited from staff members and students at the University of Tasmania, Australia, were invited to take part in the study. Recruited participants attended the university research center for a test procedure that took ≈ 10 to 15 minutes and used their mobile phones to record video and audio data. There was a convenience sampling of participants, so the range of models used represented a real‐world consecutive sample of commonly used smartphone devices.

### 2.2 Ethics and consent

The University of Tasmania Human Research Ethics Committee approved the TapTalk Project (HREC reference H0026879), which is also registered on the ClinicalTrials.gov registry (NCT 06114914). Participants gave informed consent, and all procedures were carried out in accordance with the National Health and Medical Research Council's National Statement on Ethical Conduct in Human Research and the Declaration of Helsinki.

### 2.3 Data collection

#### 2.3.1 TapTalk protocol

**FIGURE 1:**
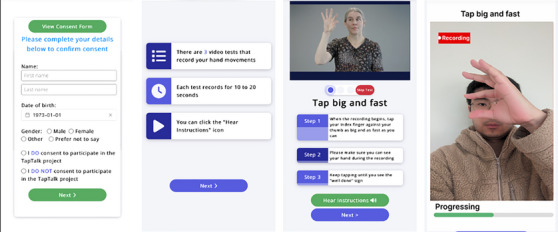
Examples of the TapTalk user interface showing a series of screenshots: (A) the consent screen, (B) the finger‐tapping tests overview instruction screen, (C) the “big and fast” test instruction screen, and (D) the "big and fast" finger‐tapping recording screen.

The TapTalk protocol comprises five tasks: three finger‐tapping tests and two speech tests (see Table 1). Each finger‐tapping task is performed with the palm visible to the camera and the tests comprise a 10 second recording of fast finger tapping (index finger tapping repetitively against the thumb), a 10 second recording of dual‐task finger tapping (index finger tapping repetitively against the thumb while counting back aloud from 100) and a 10 second recording of sequential finger tapping (index finger taps against the thumb, then the middle finger taps against the thumb, and then the ring finger against the thumb, and then the sequence is repeated in this order).^25^ The speech tests include two oral diadochokinesis (DDK) tasks: a 10 second recording of fast repetition of articulating the sound “pa‐pa‐pa…” and a 10 second recording of fast repetition of articulating the alternating sounds “pa‐ta‐ka….” Figure 1 shows example screenshots of TapTalk. Each screen of the TapTalk full protocol is enclosed in supporting information.

**TABLE 1:** TapTalk test protocol.

	Test	Description	Duration *seconds*
Finger tapping	Big and fast	Tap the dominant hand's index finger against the thumb as big and fast as possible	10
	Dual‐task	Tap the dominant hand's index finger against the thumb as big and fast as possible, while counting aloud backward from 100	10
	Sequence	Tap the dominant hand index fingertip against the thumb, then the middle finger against the thumb, ring finger against the thumb (and so on, repeating the sequence in this order) as big and fast as possible.	10
Speech	Pa‐Pa‐Pa	Say “Pa, Pa, Pa…” repeatedly as fast as possible	10
	Pa‐Ta‐Ka	Say “Pa, Ta, Ka, Pa, Ta, Ka…” repeatedly as fast as possible	10

*Note*: This table demonstrates the types of different finger‐tapping tests and speech diadochokinesis tests.

#### 2.3.2 Experiment design

A researcher (acting as a dummy participant) sat at a table with the gold‐standard data collection devices placed 25 cm in front of them—the Polhemus electromagnetic source^26^, ^27^ for movement data collection, and a high‐quality Yeti microphone (Model: 988‐000448)^28^ for audio data collection. The recruited participant sat opposite the researcher, installed the TapTalk app on their smartphone, and then placed it on a mark on the table, which was 60 cm away from the researcher's edge of the desk and 35 cm from the gold‐standard recording device. The participant used the TapTalk app to video record the researcher performing the three finger‐tapping tests and then to audio record the two speech tests.

**RESEARCH IN CONTEXT**

- **Systematic Review**: A review using PubMed and Google Scholar found smartphones are increasingly being used to collect video and audio data in research studies of neurodegenerative conditions. Motor and speech changes occur in the two most common neurodegenerative conditions—Alzheimer's disease and Parkinson's disease. However, no previous studies have validated video and audio data across a range of devices.
- **Interpretation**: We outline the protocol of our new smartphone application, TapTalk, which provides a remote, unsupervised solution for analyzing motor and speech data. We validated data extracted through 20 diverse smartphone devices: > 90.3% of video recordings, and 98.3% of audio recordings had extracted frequency measures within +/–1 Hz of the gold‐standard measures.
- **Future Directions**: This experimental study is the first to validate video and audio data collected across a range of smartphone devices. The next steps include an unsupervised validation study against established biomarkers, and diagnostic criteria, of Alzheimer's disease and Parkinson's disease, and an evaluation of usability and reliability in older adults with a range of computer literacies and cognitive function levels.

#### 2.3.3 Collection of gold‐standard hand movement and speech data for validation

To capture movement data during finger‐tapping tests, two lightweight Polhemus sensors (0.89″ in L x 0.50″ in W x 0.45″ in H, 0.13 oz.) were affixed to the researcher's right hand with one sensor on the lateral aspect of the index fingertip and the other on the dorsal aspect of the thumbtip—both secured with clear adhesive tape. The Polhemus PATRIOT system uses electromagnetic technology to deliver six degrees of freedom tracking standards at a sample frequency of 60 Hz.^29^ In this configuration, the sensors remained discreetly positioned, avoiding interference with the video analysis as they were not visible to the smartphone camera. A high‐quality Blue Yeti USB microphone (recording resolution of 48KHz/16‐bit) recorded audio data during speech‐like tests.^28^

### 2.4 Feature extraction

#### 2.4.1 Hand movement features from TapTalk video recordings

TapTalk videos were securely stored in the University of Tasmania‐protected server and analyzed offline. To extract hand movement features from videos, the key points of the fingertip and thumbtip were tracked. To do so, the computer vision tool, Google Mediapipe,^30^ was used to automatically detect the palm and then trace the index fingertip and thumbtip in videos. The hand key point tracking algorithm integrated into Google Mediapipe was executed for each frame of the video, resulting in a collection of thousands of 2D (*x, y*) coordinates representing the positions of the index fingertip and thumbtip in pixels on the frame.

**FIGURE 2:**
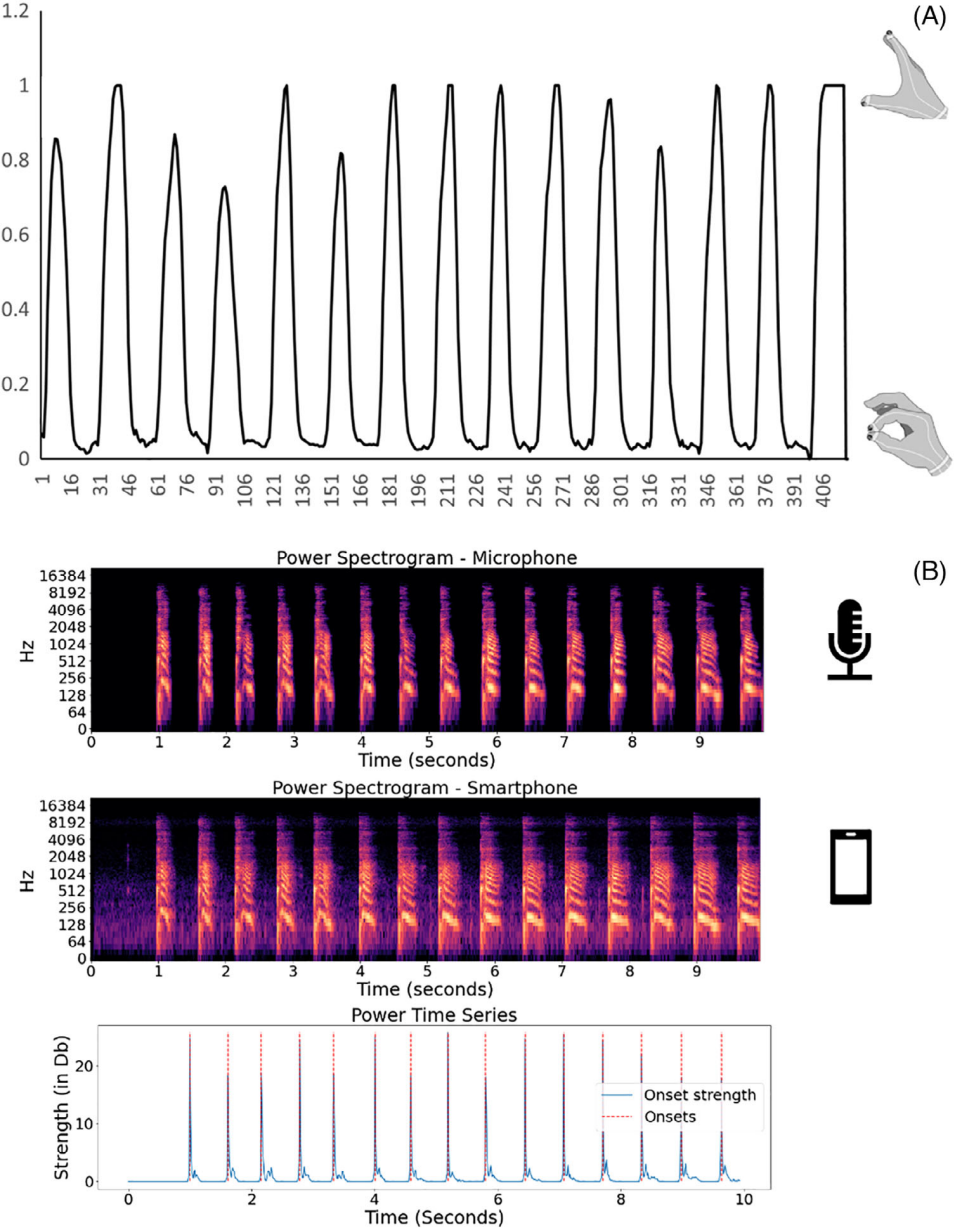
(A) Distance‐versus‐time curve. The *x* axis is the time frame, and the *y* axis is the normalized tapping amplitude. (B) Audio data processing. The top shows the power spectrogram for a typical pa‐pa‐pa test, the middle shows the power spectrogram after denoising the original audio, and the bottom shows the event detection over time.

Based on the detected key points, the displacement between the index fingertip and thumbtip, measured in pixels, was determined as the Euclidean distance for each frame of the video, using the 2D (*x, y*) coordinates of these points. Subsequently, each video produced a sequence of displacement values, which were then graphed to construct a displacement‐versus‐time curve. The *x* axis of the curve represents the time frame, while the *y* axis represents the displacement in pixels. Figure 2A visually depicts the displacement‐versus‐time curve for a specific trial captured in the video. After the generation of the displacement‐versus‐time curve, various finger‐tapping features were extracted, encompassing parameters such as speed, rhythm, and decrement. A comprehensive explanation of the extracted features is provided in Table 2, with detailed calculations outlined in the supporting information.

**TABLE 2:** Movement features and speech features.

	Feature	Description	Feature category
Finger‐tapping features	Mean tapping frequency (M‐TF)	Number of tapping cycles per second	Speed
	Coefficient of variance of tapping frequency (COV‐TF)	Variance of tapping frequency over the recording period	Rhythm
	Intra‐individual variance (IIV)	Variance of tapping cycle durations over the recording period	Rhythm
	Decrement on speed (DoS)	Decline of tapping frequency over the recording period	Decrement
Speech‐like features	Mean speaking frequency (M‐SF)	Number of syllables per second	Speed
	Coefficient of variance of speaking frequency (COV‐SF)	Variance of syllable frequency over the recording period	Rhythm
	Intra‐individual variance (IIV)	Variance of syllable durations over the recording period	Rhythm
	Decrement on speed (DoS)	Decline of syllable frequency over the recording period	Decrement

*Note*: This table demonstrates the detailed hand‐finger tapping movement features and the detailed diadochokinesis test features, along with their categories.

#### 2.4.2 Hand movement features from gold‐standard wearable sensors

The Polhemus sensors tracked the index fingertip and thumbtip and generated 3D (*x, y, z*) coordinates representing the position of each sensor (i.e., the position of the index fingertip and thumbtip) in the real world. Similar to the displacement‐versus‐time curve derived from video data, a displacement‐versus‐time curve was computed using the Euclidean distance based on the 3D coordinates. This curve was then graphed to visualize the displacement over time. After the generation of the displacement‐versus‐time curve, the finger‐tapping features listed in Table 2 were extracted, providing a consistent basis for analysis and comparison.

#### 2.4.3 Extraction of speech features from TapTalk app and gold‐standard microphone

**FIGURE 3:**
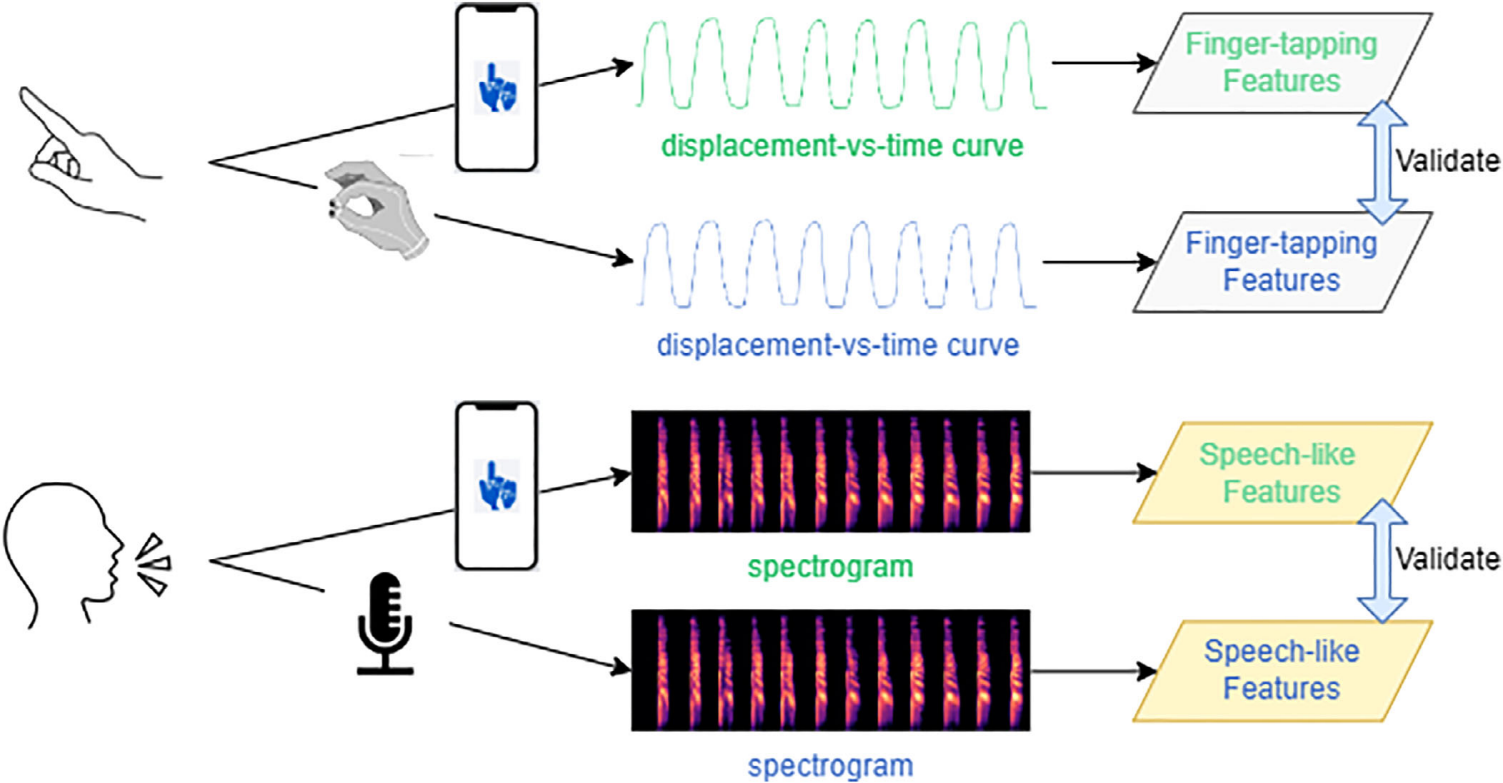
This figure visually presents the comprehensive analysis and validation process encompassing both finger‐tapping test data (gathered from a smartphone and gold‐standard device) and DDK test data (gathered from a smartphone and gold‐standard microphone). DDK, diadochokinesis.

The data processing procedure was the same for audio recordings obtained from the smartphone and the high‐quality microphone. Initially, a denoising step was applied using the Python package “noisereduce”^31^ to eliminate background noise that could potentially interfere with subsequent analyses. Subsequently, the identification of speech events (instances when the tester uttered “pa,” “ta,” or “ka”) was carried out using the Python package Librosa.^32^ This process involved extracting both the time points and the intensity of these events.^33^ The intensity values were then normalized using the min–max normalization method.^34^ Like the approach used in the finger‐tapping test, a strength‐versus‐time curve was generated. Here, the *x* axis represents time, and the *y* axis depicts the normalized speaking power. Figure 2B illustrates the whole process from denoising to getting the strength‐versus‐time curve captured by the high‐quality microphone. After producing the strength‐versus‐time curve, a set of diverse speaking features was derived, including key parameters such as speed and rhythm. For a comprehensive understanding of each extracted feature, Table 2 provides detailed descriptions. Extensive calculations for these features can be found in the supporting information, offering in‐depth insights and references for further exploration. Additionally, the entire data validation process is illustrated in Figure 3.

### 2.5 Data analysis

To ascertain the validity of data collected through TapTalk across a range of smartphone devices, we conducted a comparative analysis between the TapTalk set and the gold‐standard set of features using paired Welch *t* tests^35^ and Bland–Altman analysis.^36^ In the Welch *t* test, a *P* value > 0.05 suggests any differences between the two sets of features are non‐significant. The Scipy.stats^37^ function from the Python package was used.

## 3 RESULTS

Thirty‐one participants completed TapTalk. Two participants could not complete the test due to a network error. After excluding a small number of samples due to poor data quality from the movement sensors (gold‐standard method), there were 72 video‐Polhemus pairs of “Tap” test data and 58 smartphone‐microphone pairs of “Talk” test data included in the data analysis. No samples were excluded due to poor‐quality data from the smartphone TapTalk app. Specifically, this comprised 29 + 24 + 19 pairs for “Tap” tests 1, 2 and 3, respectively, and 29 + 29 pairs for “Talk” tests 1 and 2, respectively.

These data comprised a convenience sample of 20 different smartphones: 11 iPhones and 9 Androids, with specific devices and number of participants with each device listed in Table 3. There were 21,600 video frames of hand movement data (from 72 videos, recorded by 20 different devices × 10 second duration each × 30 frames‐per‐second (fps) and 58,000 Msecs of audio recordings of speech data (from 29 audio recordings by 20 different devices × 2 tests × 1000 Msecs of 16‐bit/44.1 kHz) included in the analysis.

**TABLE 3:** Different mobile phones were used in the study.

iPhone	Number of users	Android	Number of users
iPhone 15 Pro Max	1	Google Pixel 3	1
iPhone 14 Pro Max	2	Google Pixel 4A	4
iPhone 14	1	Google Pixel 6 Pro	1
iPhone 13 Pro Max	4	Samsung Flip 3	1
iPhone 13	3	Samsung Galaxy S21	1
iPhone 12	1	Samsung Galaxy A71	1
iPhone 11	2	Samsung Galaxy S20	1
iPhone SE	1	Samsung S23 Ultra 5G	1
iPhone X	2	Nokia G50	1
iPhone XS	1		
iPhone 8 Plus	1		

*Note*: This table demonstrates the 20 different devices used in the study, which generated the 72 sets of video data and the 29 sets of audio data included in the analysis.

All finger‐tapping features (speed, rhythm, and decrement related) extracted from the videos and all speech features extracted from the audio recordings were validated against the gold standard. Specifically, 90.3% of smartphone video‐recorded motor frequencies and 98.3% of audio‐recorded speech frequencies were within ± 1 Hz of the gold standard. For intra‐individual variances, 100% of smartphone video recordings and 100% of audio recordings were within ± 1 Hz of the gold standard; for variations in frequency, these figures were 100% and 100%, respectively. For decrements in speed, 100% of video‐recorded motor data and 100% of audio‐recorded speech data were within ± 1 Hz of the gold‐standard measures (see Bland–Altman plots in Figure 4).

FIGURE 4Bland–Altman plots of four different finger tapping features for big and fast finger tapping test (A), dual‐task finger tapping test (B), and sequence finger tapping test (C) with the borders from −1.96 standard deviation to +1.96 standard deviation (green dashed lines) and the mean difference (red dashed line).

**Figure:**
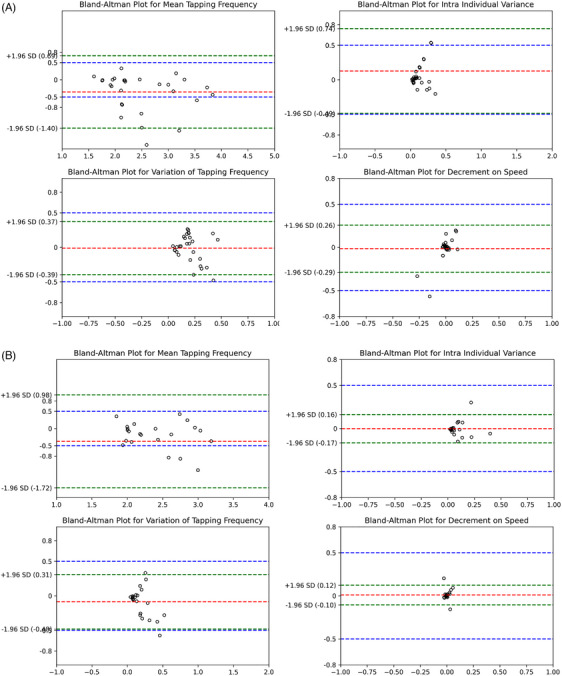

**Figure:**
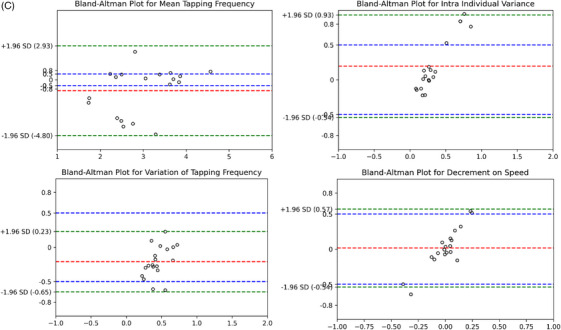

The range of finger‐tapping frequencies for iPhone was 1.76 to 4.05 Hz with a mean (standard deviation [SD]) of 2.55 (0.67) Hz and for Android was 1.55 to 3.84 Hz with a mean (SD) of 2.70 (0.81) Hz. The range of speech frequencies for iPhone was 2.24 to 3.87 Hz, mean of 2.96 (0.47) Hz and for Android was 2.02 to 4.23 Hz, mean of 3.11 (0.70) Hz. In summary, we found that there was no difference between the hand motor, and speech, data collected through TapTalk and the gold‐standard recording devices (see Table 4).

**TABLE 4:** Paired Welch *t* test results for three different finger‐tapping tests and two different speech tests.

	Feature	Big and fast	Dual task	Sequence
Finger tapping	Frequency	0.0535	0.0824	0.0538
	Variation of frequency	0.7809	0.0792	0.0513
	Intra‐individual variance	0.0515	0.9534	0.0560
	Decrement on speed	0.5633	0.3625	0.8171
	**Feature**	**Pa‐Pa‐Pa**	**Pa‐Ta‐Ka**	
Speech‐like	Frequency	0.0574	0.3442	
	Variation of frequency	0.2407	0.0542	
	Intra‐individual variance	0.1018	0.0633	
	Decrement on speed	0.2177	0.0545	

*Note*: This table demonstrates the validation results between finger‐tapping test features extracted from  smartphone video data and finger‐tapping test features extracted from the gold‐standard device (Polhemus sensors); and the validation results between diadochokinesis (DDK) test features extracted from smartphone audio data and DDK test features extracted from the gold‐standard device (Blue Yeti USB microphone). *P* values are presented, where *P* > 0.05 implies smartphone measures are not significantly different to the gold‐standard movement sensor and high‐quality microphone data.

## 4 DISCUSSION

We have described and validated a 2 minute protocol for a new smartphone app, TapTalk, across 20 different smartphones. We have compared the motor and speech features extracted from the video and audio smartphone data against gold‐standard high‐quality movement and audio‐recording measures. Notably, this is the first study to validate video and audio data collection across such a wide range of smartphones and this addresses an important gap for all studies that use smartphones to collect video and/or audio data in research.

Digital technology offers a means to evaluate the subtle cognitive decline characteristics of preclinical AD, as confirmed by biomarkers. Validation studies for such digital technologies have primarily been carried out on established platforms such as PCs and tablets, with limited exploration on more innovative platforms like smartphones.^6^ In video‐ and audio‐based smartphone applications, no studies have been found that use smartphones for movement video analysis in AD. Only one previous study has validated the efficacy of smartphone applications for collecting audio data and calculating features in DDK tests. Kadambi et al. collected DDK test data from 82 people with amyotrophic lateral sclerosis and 26 controls using smartphones and compared these DDK data to clinical scores provided by trained annotators.^38^ The algorithm estimated DDK frequencies were highly correlated with manual annotations (*r* = 0.98) and the estimated frequencies achieved a high test–retest reliability (*r* = 0.95). However, the source of audio data in their study, originating from smartphones, introduces potential validity concerns because the audio from smartphones has not been validated against gold‐standard audios. The gold standard was derived from clinical raters who are known to have bias and variability. It would be better to compare the audio data from smartphones to gold‐standard recording devices (i.e., compare one objective measure to another objective measure). Moreover, the types of smartphones used in the analysis were not specified, which may also affect the results. In contrast, our study is the first to compare data across a much wider range of smartphone devices and compare to a gold‐standard objective measure for both movement‐based (finger tapping test) and speech‐based (DDK) tests. This comprehensive approach not only addresses the limitations of prior work but also sets a new precedent for smartphone‐based assessment methodologies. In a speech research study by Illner et al.,^39^ 60 monologues (30 from patients with PD and 30 from healthy controls) were collected using the Sony Xperia Z1 Compact (Android system). The monopitch feature extracted from these monologues demonstrated robustness in distinguishing between the PD and control groups, particularly when the signal‐to‐noise ratio was < 6 dB (*P* < 0.001). However, this study was only validated against a single type of Android smartphone, and microphone quality can vary among different smartphone models due to the robustness against background noise. In our investigation, we used 20 diverse smartphone models to validate speech features, resulting in a more comprehensive and robust validation study.

With the TapTalk smartphone application proving to be a valid tool for extracting features in both “Tap” and “Talk” tests, the potential to transform research in detecting early stages of AD, PD, or other neurological conditions becomes evident. This development opens avenues for conducting large‐scale studies remotely, in which participants can conveniently contribute data from the comfort of their homes. The accessibility and ease of use of smartphone applications can significantly enhance participant engagement and reduce barriers to participation, ultimately contributing to a more comprehensive understanding of neurodegenerative conditions. This study underscores the transformative impact that smartphone technology can have on research methodologies and emphasizes the potential for future advancements in remote health‐care monitoring and diagnosis.^40^

Our study boasts several notable strengths that contribute to its comprehensive and robust nature. First, we encompassed a wide range of smartphones in our investigation, ensuring the applicability and generalizability of our findings across various devices. This is the first to validate motor and speech (DDK) data using different mobile devices; previous studies have not analyzed the different smartphone variabilities on the data analysis results. Second, unlike some previous studies that focused solely on one finger‐tapping or one speech test, we conducted several different tests for both finger tapping and speech, broadening the scope of our analysis and providing a more holistic understanding of mobile‐based assessment capabilities. Moreover, by involving real‐life participants with a convenience sample of current smartphones rather than solely relying on researchers, we ensured the relevance and authenticity of our data. Additionally, we rigorously extracted numerous features from both hand and speech tests, enriching the depth of our analysis and enabling comprehensive insights into participant performance as multimodal data becomes more important in the detection and monitoring of neurodegenerative disease.^18^ Last, our study used robust gold‐standard measures, enhancing the strength and accuracy of our findings, thus solidifying the credibility of our research outcomes.

Several limitations also need to be considered. First, there is a range of other smartphones not tested and the focus on specific assessments, such as finger tapping and speech (syllabic) production are just two broad groups of tests that do not encompass wider neurodegenerative symptomatology. Also, the validation was only assessed at ≈ 60 cm, as this was felt to replicate a typical arm length, but it would be important to validate with other variables such as lighting, range of frequency of movements, and background noise. Additionally, some *P* values in our study are close to 0.05, suggesting that the study might have been underpowered to detect significant differences between smartphone and gold‐standard sensors. Future research with larger cohorts is recommended to confirm our findings and provide a more definitive assessment. Finally, this pilot study's validation was limited to 31 healthy adults using 20 different smartphone models. The findings may not fully reflect the app's performance in clinical populations, such as individuals with AD or PD, who may exhibit greater motor and speech variability that could impact the app's validity. These limitations underscore the need for ongoing research to refine and expand the utility of TapTalk in neurodegenerative research contexts.

Future research stands to gain from broadening participant recruitment to encompass a larger and more diverse pool, including clinical samples with AD or PD, thereby augmenting the generalizability of findings. This inclusive approach will empower researchers to explore multiple facets of the investigated topic, unveiling fresh insights into the potential of smartphone technology to advance research methodologies. Further exploration of smartphone application data collection in research warrants the integration of a wider array of assessments, including additional motor tests such as gait analysis. Moreover, investigating the clinical applicability and correlation with established diagnostic criteria presents promising avenues for future exploration.

This study not only provides valuable insights into the validity of using smartphones for research purposes but also introduces a promising tool, TapTalk, for remotely collecting motor and speech (DDK) data. With the growing interest in using smartphones for research, the findings from this study offer crucial information on the rigor and validity of various measures conducted through smartphone applications. Moreover, TapTalk presents an innovative solution for researchers seeking to gather motor and speech data remotely, as it is self‐administered and brief, filling a current gap in available tools. However, before widespread implementation, it is essential to undertake further studies to assess the reliability and usability of TapTalk in an unsupervised environment. Once established, TapTalk has the potential to revolutionize data collection methods in research settings, particularly for remote and large‐scale studies of AD and PD.

## CONFLICT OF INTEREST STATEMENT

The authors declare no conflicts of interest. Author disclosures are available in the supporting information.

## CONSENT STATEMENT

All human subjects provided informed consent.

## Supporting information

Supporting information

Supporting information

## Data Availability

The data supporting the findings of this study are available on request from the corresponding author. The data are not publicly available due to privacy or ethical restrictions.
