# Supplementary material for: Smartphone automated motor and speech analysis for early detection of Alzheimer's disease and Parkinson's disease: Validation of TapTalk across 20 different devices
Source: Alzheimers Dement (Amst). 2024 Oct 23;16(4):e70025. doi: 10.1002/dad2.70025 (PMC11496774; doi:10.1002/dad2.70025)

**Supplementary Document**

1. **Extraction of movement features from the video recordings**

The following equations outline the details of how different **finger tapping** movement features are calculated from the video-recorded hand movement data for Mean Tapping Frequency (M-TF), Coefficient of Variance of Tapping Frequency (COV-TF), Intra Individual Variance (IIV), Decrement on Speed (DoS).

|  | $\text{M-TF = }\frac{1}{K_{p}-1}\sum_{k=2}^{K_{p}} \frac{1}{t_{(k)}-t_{(k-1)}}$ | (1) |
| --- | --- | --- |
|  | $\text{COV-TF}= \sqrt{\frac{\sum_{k=2}^{K_{p}} \left( \frac{1}{t_{\left( k \right)}-t_{\left( k-1 \right)}}-\text{M-TF} \right)^{2}}{K_{p}-1}}/\text{M-TF}$ | (2) |
|  | $IIV= \sqrt{\frac{1}{K_{p}-1}\sum_{k=2}^{K_{p}} {\left[ {(t}_{\left( k \right)}-t_{\left( k-1 \right)} \right)-\text{M-ITI}]}^{2}}$ | (3) |
|  | $\text{DoS}= \frac{1}{K_{p}-1}ln(\frac{1}{t_{\left( 2 \right)}-t_{\left( 1 \right)}}/\frac{1}{t_{\left( K_{p} \right)}-t_{(K_{p}-1)}})$ | (4) |

$K_{p}$ refers to the number of peaks, $K_{v}$ refers to the number of valleys, $t_{k}$ refers to the time point at $k^{th}$ peak and $a_{k}$ refers to the normalized amplitude.

**(ii) Extraction of speech-like features from the audio recordings**

The following equations outline the details of how different features are calculated from the audio-recorded speech-like data for Mean Speaking Frequency (M-SF), Mean Inter Tapping Interval (M-ITI), Coefficient of Variance of Speaking Frequency (COV-SF) and Intra Individual Variance (IIV).

|  | $\text{M-SF = }\frac{1}{K_{p}-1}\sum_{k=2}^{K_{p}} \frac{1}{t_{(k)}-t_{(k-1)}}$ | (1) |
| --- | --- | --- |
|  | $\text{COV-SF}= \sqrt{\frac{\sum_{k=2}^{K_{p}} \left( \frac{1}{t_{\left( k \right)}-t_{\left( k-1 \right)}}-\text{M-TF} \right)^{2}}{K_{p}-1}}/\text{M-TF}$ | (2) |
|  | $IIV= \sqrt{\frac{1}{K_{p}-1}\sum_{k=2}^{K_{p}} {\left[ {(t}_{\left( k \right)}-t_{\left( k-1 \right)} \right)-\text{M-ITI}]}^{2}}$ | (3) |
|  | $\text{DoS}= \frac{1}{K_{p}-1}ln(\frac{1}{t_{\left( 2 \right)}-t_{\left( 1 \right)}}/\frac{1}{t_{\left( K_{p} \right)}-t_{(K_{p}-1)}})$ | (4) |

$K_{p}$ refers to the number of speaking events, $t_{k}$ refers to the time point at $k^{th}$ event.

**(iii) Examples of data from the wearable Polhemus movement sensors**

The following charts provides examples of poor-quality displacement-vs-time curves from the Polhemus sensors during a finger tapping task and an example of a standard/good quality recording for comparison. The X-axis is the time frame, and the Y-axis is the displacement between index fingertip and thumb-tip.

**Standard/good quality recording**


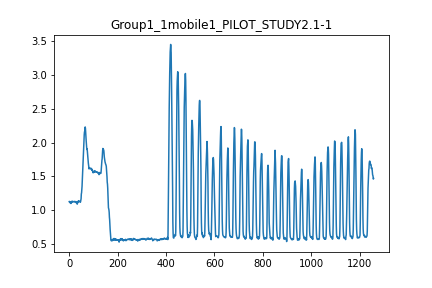


**Examples of poor-quality recordings** (excluded from analysis):


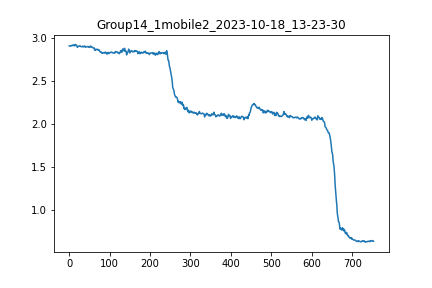

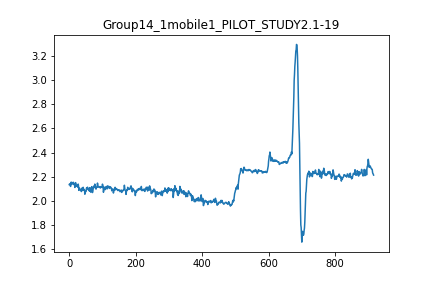


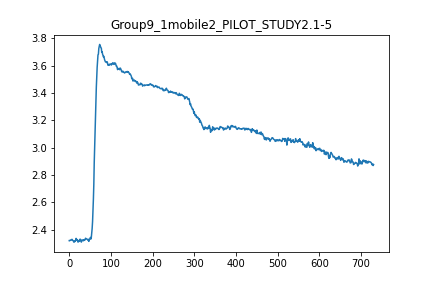

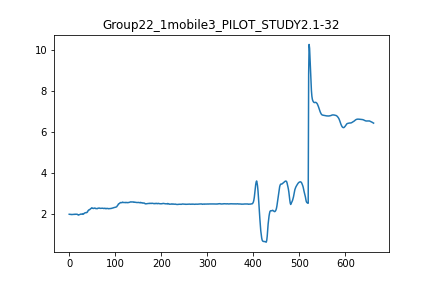

Supplement: Supplementary file 1 — Supporting information [file DAD2-16-e70025-s002.docx]
